# Longitudinal Alterations of Alpha-Synuclein, Amyloid Beta, Total, and Phosphorylated Tau in Cerebrospinal Fluid and Correlations Between Their Changes in Parkinson's Disease

**DOI:** 10.3389/fneur.2018.00560

**Published:** 2018-07-11

**Authors:** Mahsa Dolatshahi, Shayan Pourmirbabaei, Aida Kamalian, Amir Ashraf-Ganjouei, Mehdi Yaseri, Mohammad H. Aarabi

**Affiliations:** ^1^Faculty of Medicine, Tehran University of Medical Sciences, Tehran, Iran; ^2^Department of Epidemiology and Biostatistics, School of Public Health, Tehran University of Medical Sciences, Tehran, Iran

**Keywords:** Parkinson's disease, Cerebrospinal fluid (CSF), Longitudinal, alpha-synuclein, tau, beta-amyloid

## Abstract

**Background:** Parkinson's disease (PD) is characterized by proteinopathies and these proteinopathies seem to interact synergistically and lead to protein aggregations and changes in protein cerebrospinal fluid (CSF) levels. In this study, we aimed to explore the longitudinal changes of CSF a lpha-synuclein (α-syn), total tau (t-tau), phosphorylated tau (p-tau), and beta-amyloid (Aβ_1−42_) and their relationships with each other and with baseline clinical entities like REM sleep behavior disorder (RBD), cognitive impairment, motor symptoms, and olfaction dysfunction.

**Method:** One hundred and twelve non-demented PD patients and 110 controls were recruited from Parkinson's Progression Markers Initiative (PPMI).We used a linear mixed model within groups to assess longitudinal protein changes over 6 and 12 months and a random regression coefficient within the linear mixed model to investigate the correlation between proteins and their baseline clinical characteristics.

**Results:** P-tau was lower in PDs only at baseline, but during a year, p-tau increased more rapidly in PDs than controls. Aβ_1−42_ was not significantly different between groups at any separate timepoint; however, when assessed longitudinally, Aβ_1−42_ showed significant changes in both groups. Conversely, t-tau and α-syn differed significantly between groups, but their longitudinal changes were not significant in either of the groups. Moreover, all proteins' baseline levels, except p-tau, could determine estimated longitudinal tau changes. Baseline RBDSQ scores but not UPDRS III, MoCA, or UPSIT scores were predictive of longitudinal increase in α-syn levels.

**Conclusion:** Longitudinal changes in levels of CSF proteins are related to each other and could help researchers further understand PD pathology. In addition, RBD seems to be a potential prognostic factor for PD progression. However, in order to reach a consensus, longer follow-up times are required.

## Introduction

As thelife expectancy of population increases, neurodegenerative diseases like Parkinson's disease (PD) become the spotlight in medicine (1). PD is characterized by several pathological mechanisms like lysosomal damage, oxidative stress, and most importantly protein aggregation. The main form of proteinopathy in PD has long been considered to be synucleinopathy, i.e., intraneuronal inclusions of alpha-synuclein in the form of Lewy bodies and Lewy neurites. However, recent research has indicated the accompanying role of other AD-associated proteinopathies like tauopathy and beta-amyloidopathy (2).

Moreover, it is known that PD symptoms progress heterogeneously (3) which can be mainly attributed to the variations in the predominant pathological mechanisms (4, 5). These variations can make a great difference in manifestations of PD symptoms such as cognitive dysfunction and contribute to disease heterogeneity. For example, concomitant AD-pathology (tau and beta-amyloid aggragations) is a more common observation in PD dementia (PDD) (6, 7). On the other hand, recently it has been revealed that the predominant clinical manifestations which patients are presented with at baseline might be a predictor for progression of disease, both from a pathophysiological and clinical point of view. More importantly, these differences in the type and severity of underlying proteinopathies determine the most effective therapeutic procedure for each person, which is the cornerstone of personalized medicine. Thus, clinicians would benefit from a method to detect the main pathological pathways leading to dopaminergic neuron loss and PD symptoms in each individual, in association with their different clinical entities, for both diagnostic and curative purposes (8, 9).

On the other hand, many studies have reached the notion that mutual interactions between different proteins promote the aggregation of these proteins *in vivo* and accelerate cognitive dysfunction (10). Most notably, alpha-synuclein induces polymerization, and aggregation of tau and thereby promotes the formation of intracellular amyloid–tau inclusions (11, 12). It also engages in cross-seeding with tau proteins so they tend to co-exist in intracellular inclusions (13).

Importantly, these proteinopathies are mirrored in changes of CSF protein concentrations, which can be easily measured in clinic. Unfortunately, despite the large number of studies, the diagnostic utility of CSF biomarkers has been unsatisfactory (14).

Numerous studies have assessed the concentration of CSF biomarkers in PD. However, most of these studies were cross-sectional and just a few studies assessed longitudinal changes of CSF markers and lead to inconsistant results. These cross-sectional studies have mostly shown decreased CSF levels of alpha-synuclein (α-syn) in PD and other synucleinopathies compared to healthy controls (15–20). In addition, the levels of Alzheimer's disease (AD) biomarkers such as total tau (t-tau), phosphorylated tau (p-tau), and beta-amyloid 1-42 (Aβ_1−42_) have been previously shown to be decreased or normal in PD patients (17–21). Apart from the mentioned findings, there has been some different observations. For example, in the study conducted by Montine et al., one third of non-demented PD patients and half of PDD patients showed higher CSF tau levels compared to healthy controls (17). Moreover, the study of Parkinson Progression Markers Initiative (PPMI) (200 healthy controls and 400 PDs) showed non-significantly changed levels of Aβ_1−42_ compared to controls at baseline (20). Specific patterns of changes in levels of these proteins in different groups of PD patients with various PD symptoms have been observed, which are reviewed elsewhere (22, 23).

Longitudinal studies that evaluated longitudinal changes of CSF proteins (α-syn, t-tau, p-tau, and Aβ_1−42_) levels in PD patients have reached heterogeneous results e.g., the study conducted by Hall et al. has shown significantly increasing levels of all proteins except Aβ_1−42_ during a two-year follow-up, in which increase of p-tau was associated with motor symptoms aggravation and cognitive decline (21). Another study done by Majbour et al. revealed no significant change in levels of t-tau, p-tau, Aβ_40_, and Aβ_42_ but an increase in total and oligomeric α-syn levels and a decrease in serine129 phosphorylated α-syn during a two-year follow-up (24). The study conducted by Stewart et al. has shown a decline in alpha-synuclein over a 2-year follow-up in patients of DATATOP cohort who received medication, which was associated with an improvement in cognitive abilities but was not correlated with motor symptoms (25). On the other hand, a recent study by Forland et al. showed nonsignificant changes of CSF alpha-synuclein in a 4-year follow-up (26). Another study done by Boungiorno et al. has shown a longitudinal decline in CSF Aβ but an increase in t-tau levels during 18 months, which were not significantly associated with cognitive decline (27). On the whole, these studies have shown quite inconsistent results regarding longitudinal changes of CSF proteins.

Thus, it seems that conducting studies with a large sample size in ongoing cohorts with drug-naïve cases like PPMI can be helpful in interpretation of these heterogeneous results and complementing our knowledge about this cohort. Furthermore, investigating the correlations between baseline levels of proteins and their changes in association with clinical manifestations facilitates estimation of patients' prognoses and can lead to a better understanding of PD pathogenesis.

In this study, we used a relatively large sample size in recent-onset, drug-naive, non-demented patients recruited from PPMI cohort to investigate the longitudinal changes in CSF protein levels and also their association with each other, with baseline levels of each protein, and also the clinical entities. These longitudinal assessments might elucidate the interaction between pathological mechanisms during disease progression and whether they have any associations with heterogeneous clinical manifestations.

## Methods

### Participants

The participants of this study were recruited from the Parkinson Progression Markers Initiative (PPMI) database freely available at http://www.ppmi-info.org/. PPMI cohort comprises 400 recently diagnosed PD and 200 healthy subjects followed longitudinally for biomarker assessment at 21 clinical sites using standard data acquisition protocols (28). The PPMI study was approved by the Institutional Review Board of all participating sites and all participants were given written informed consent before inclusion in the study. PD subjects of PPMI study were recruited at disease threshold, meaning that they had been diagnosed within 2 years while they were drug-naïve at baseline. The clinical criteria for PD diagnosis included asymmetric resting tremor and/or asymmetric bradykinesia. In addition, all subjects underwent dopamine transporter (DAT) imaging and DAT deficit was considered necessary for PD diagnosis. The subjects were assessed for motor symptoms using Unified Parkinson's Disease Rating Scale part III (UPDRS III), cognitive impairment using Montreal Cognitive Assessment (MoCA). REM sleep Behavior Disorder (RBD) and olfaction dysfunction were assessed using REM Sleep Behavior Disorder Screening Questionnaire (RBDSQ) and University of Pennsylvania Smell Identification Test (UPSIT), respectively. Healthy subjects were required to have no significant neurologic dysfunction, no first-degree family member with PD and MoCA >26 (2011). In PPMI cohort longitudinal data for CSF samples were available only for a portion of the patients. Thus, we selected the subjects for which longitudinal CSF data were available at baseline (BL), the second visit (V02) i.e., 6 months after recruitment, and the fourth visit (V04) i.e., one year after recruitment. We excluded the patients who met the criteria for PD dementia (PDD) at baseline and those who had recently received medications to have a clinically homogenous sample. In the end, 112 PD patients and 110 matched healthy subjects with the above-mentioned properties were included in the study. Complementary data on disease duration, age, gender, and UPDRS III score at different time points is provided in Supplementary Tables 1–4.

### CSF samples collection and analysis

CSF was collected by using standardized lumbar puncture procedures. CSF was collected into siliconized polypropylene tubes and the first 1–2 mL of CSF of sent to the site's local laboratory for routine testing for cell count, total protein level, and glucose level. An additional 15–20 mL of CSF was transferred into 15-mL conical propylene tubes at room temperature, mixed gently, centrifuged at 2,000 g for 10 min at room temperature, and transferred into 1.5-mL pre-cooled siliconized polypropylene aliquot tubes followed by immediate freezing on dry ice. The frozen aliquots of CSF were shipped overnight to the PPMI Biorepository Core Laboratories on dry ice and then thawed, aliquoted into 0.5-mL siliconized polypropylene tubes, refrozen once, and stored at −80°C. The coded frozen aliquots of CSF were transferred from the PPMI Biorepository Core laboratories to the University of Pennsylvania and to Covance for analyses. CSF Aβ1-42, t-tau and p-tau were measured using the xMAP-Luminex platform with INNOBIA AlzBio3 immunoassay kit-based reagents (Fujirebio-Innogenetics, Ghent, Belgium). CSF α-syn and CSF hemoglobin levels were analyzed using appropriate commercially available sandwich type ELISA kits (Covance, Dedham, MA) [2011; (19, 20)].

### Statistical analysis

We used Kolmogorov-Smirnov test as well as a Q-Q plot to check for the normal distribution of data. To present data, we used mean, standard deviation, median, and range. To find the difference between the two groups during the study period, we used the Mann-Whitney test. To assess the changes within each group (PD, controls) considering the correlation of measurements (CSF protein concentrations, age, disease duration), we used a linear mixed model within each group. In this analysis, multiple comparison corrections were performed by Bonferroni method. Other linear mixed models with the interaction of time and groups were used to test the difference of trends in parameters during the study course between the two groups. Correlation of different parameters and their changes during the study course was assessed with Pearson correlation. To adjust for the probable confounding effect of age, sex, and disease duration (in PD group) we used partial correlation coefficient. Finally, to evaluate the effect of baseline values of biomarkers on their own changes and the changes of other biomarkers, we used a two-step procedure: in the first step, we obtained the estimated mean change of each parameter on each subject with the use of random regression coefficients of a linear mixed model. In the next step, we used correlation coefficient to evaluate the relation of these estimations with baseline values of all parameters. Also, the correlation of different biomarkers' estimated changes was calculated. All statistical analyses were performed by R (R Foundation for Statistical Computing, Vienna, Austria), http://www.R-project.org/. A *p*-value of < 0.05 was considered statistically significant.

## Results

### Demographics

Demographics and clinical scores of study participants and their comparison are given in Table 1. UPDRS III, MoCA, and UPSIT scores were significantly lower in PD compared to controls at baseline and after a year (for MoCA and UPDRS III). MoCA and UPDRS III scores were significantly lower at V04 compared to baseline in PD patients. However, in controls, only MoCA scores were lower at V04 compared to baseline. RBDSQ scores, however, not only showed no difference between PD and control groups, but also they were not different at baseline compared to V04.

**Table 1:** Demographics and clinical tests including Unified Parkinson's Disease Rating Scale part III (UPDRS III), Montreal Cognitive Assessment (MoCA), REM sleep Behavior Disorder Questionnaire (RBDSQ), and University of Pennsylvania Smell Identification Test (UPSIT) scores at Baseline (BL) and after a year, visit 04 (V04) in Parkinson's disease (PD) and control subjects.

	**PD (*n* = 112)**	**Control (*n* = 110)**	***P*-value (PD, control) [two-sample *t*-test]**	***P*-value (BL, V04) [paired sample *t*-test]**
Age (years) (mean ± SD)	60.89 ± 10.46	61.54 ± 10.03	0.6381^a^	–
Gender (Male Number/ %)	80 M/71.42%	69 M/62.72%	0.2465^b^	–
Disease duration (months) (mean ± SD)	14.23 ± 14.08	–	–	–
UPDRS III (BL) (mean ± SD)	19.15 ± 8.16	2.41 ± 1.39	0.0000^a^	0.0027^c^(*PD*)
UPDRS III (V04) (mean ± SD)	22.98 ± 10.52	3.12 ± 1.92	0.0000^a^	0.1555^c^(*control*)
MoCA (BL) (mean ± SD)	27.03 ± 2.10	28.22 ± 1.06	0.0000^a^	0.0031^c^(*PD*)
MoCA (V04) (mean ± SD)	26.34 ± 2.42	27.29 ± 2.10	0.0023^a^	0.0000^c^(*control*)
RBDSQ (BL) (mean ± SD)	4.17 ± 2.89	3.95 ± 2.89	0.5358^a^	0.3394^c^(*PD*)
RBDSQ (V04) (mean ± SD)	3.99 ± 2.82	3.60 ± 2.99	0.6371^a^	0.5694^c^(*control*)
UPSIT baseline (mean ± SD)	20.77 ± 8.03	33.99 ± 4.69	0.0000^a^	–

*n, number; SD, standard deviation*.

a *p-values obtained by independent samples t-test*.

b *p-values obtained by chi-square independence test*.

c *p-values obtained by paired samples t-test (difference between clinical scores at baseline and after 1-year follow-up)*.

On the whole, age and disease duration were correlated with levels of some CSF biomarkers at different time points in PD and controls.

Values of Aβ_1−42_ were correlated with age in the control group and levels of t-tau showed correlation with age in PD group at all time points. Percentage of changes in levels of t-tau and Aβ_1−42_ were correlated with age in both groups.

Baseline values of p-tau and t-tau in PD groups were correlated with disease duration. Also, percentage of changes of p-tau and Aβ_1−42_ during 6 months was correlated with disease duration. Percentage of changes during one year, only for t-tau and Aβ_1−42_, was correlated with disease duration.

### Difference between PD and control in biomarker levels at different time points

The levels of α-syn and t-tau were significantly lower in PD group than controls at baseline, after six months, and after one year. P-tau level was significantly lower in PD group at baseline, but not after 6 months and one year. Levels of Aβ_1−42_ showed no significant difference between the two groups at any time point. (Table 2).

**Table 2:** Biomarker changes in PD and control group within a linear mixed model.

**Parameter**	**Control**		**PD**		**Group difference**	**P of trend with interaction of time and group**
	**Mean ± SD**	**Median (range)**	**Mean ± SD**	**Median (range)**	***P^†^***	**diff^$^**
α-synuclein						0.950
Value at baseline	2,163 ± 976	1,950 (593 to 5,238)	1,783 ± 700	1,704 (333 to 5,174)	0.007	
Value at 6 months	2,196 ± 924	2,070 (659 to 5,209)	1,822 ± 700	1,770 (680 to 4,659)	0.001	
Crude change till 6 months	33 ± 623	7 (-1,913 to 2,258)	40 ± 317	34 (−674 to 837)	0.948	
Change% till 6 months	8 ± 37	0 (−61 to 257)	6 ± 27	2 (−40 to 192)	0.943	
*P*^§^-within linear mixed model till 6 months	1.000		0.774			
Value at 1 year	2,166 ± 964	2,037 (729 to 5,295)	1,812 ± 712	1,700 (782 to 4,633)	0.004	
Crude change till 1 year	3 ± 589	−5 (−2,292 to 1,748)	30 ± 406	11 (−1,612 to 1,284)	0.872	
Change% till 1 year	5 ± 34	0 (−55 to 161)	6 ± 31	1 (−46 to 202)	0.737	
P^§^-within linear mixed model till 1 year	1.000		1.000			
P-tau						0.654
Value at baseline	17 ± 9	14 (6 to 59)	15 ± 8	12 (6 to 40)	0.032	
Value at 6 months	16 ± 8	13 (6 to 53)	15 ± 9	11 (5 to 56)	0.221	
Crude change till 6 months	−1 ± 9	0 (−24 to 39)	0 ± 10	0 (−22 to 40)	0.421	
Change% till 6 months	0 ± 0	0 (−2 to 1)	0 ± 1	0 (−2 to 2)	0.433	
P^§^-within linear mixed model till 6 months	0.983		1.000			
Value at 1 year	20 ± 13	16 (6 to 90)	19 ± 11	15 (5 to 58)	0.622	
Crude change till 1 year	3 ± 15	1 (−30 to 75)	4 ± 13	3 (−27 to 47)	0.365	
Change% till 1 year	0 ± 1	0 (−2 to 4)	0 ± 1	0 (−2 to 3)	0.256	
P^§^-within linear mixed model till 1 year	0.094		0.003			
T-tau						0.240
Value at baseline	52 ± 26	45 (18 to 188)	45 ± 18	39 (22 to 121)	0.047	
Value at 6 months	51 ± 24	45 (17 to 181)	43 ± 18	37 (16 to 135)	0.002	
Crude change till 6 months	−1 ± 9	0 (−27 to 35)	−2 ± 6	−2 (−15 to 22)	0.037	
Change% till 6 months	0 ± 0	0 (−1 to 2)	0 ± 0	0 (−2 to 2)	0.006	
P^§^-within linear mixed model till 6 months	1.000		0.007			
Value at 1 year	53 ± 27	45 (19 to 216)	44 ± 18	39 (19 to 129)	0.002	
Crude change till 1 year	1 ± 10	1 (−28 to 40)	−1 ± 6	−1 (−28 to 15)	0.047	
Change% till 1 year	0 ± 0	0 (−1 to 3)	0 ± 0	0 (−1 to 2)	0.025	
P^§^-within linear mixed model till 1 year	0.893		0.459			
Aβ_1−42_						0.785
Value at baseline	368 ± 103	379 (89 to 680)	362 ± 84	368 (140 to 627)	0.561	
Value at 6 months	374 ± 100	374 (98 to 610)	364 ± 95	374 (129 to 687)	0.245	
Crude change till 6 months	6 ± 54	5 (−230 to 152)	2 ± 52	4 (−122 to 205)	0.404	
Change% till 6 months	0 ± 3	0 (−18 to 8)	0 ± 4	0 (−28 to 19)	0.484	
P^§^-within linear mixed model till 6 months	0.835		1.000			
Value at 1 year	388 ± 106	392 (95 to 691)	382 ± 105	379 (144 to 733)	0.376	
Crude change till 1 year	20 ± 64	20 (−265 to 190)	20 ± 71	6 (−161 to 317)	0.370	
Change% till 1 year	1 ± 4	1 (−12 to 16)	1 ± 4	0 (−8 to 14)	0.428	
P^§^-within linear mixed model till 1 year	0.022		0.043			

† *P-value of comparing the groups at each timepoint based on Mann-Whitney test; however, the 95% Confidence Intervals are based on t-statistics*.

§ *Longitudinal changes within the liner mixed model in each group, multiple comparison correction performed by Bonferroni method*.

$ *Comparison between the trend of changes in groups based on interaction analysis of time and group, within a linear mixed model*.

### Longitudinal changes over 6 months and one year in CSF biomarker levels in PD and control groups

The levels of α-syn showed no significant change after 6 months and one year in neither of the groups. P-tau level had not significantly changed after 6 months in either group; however, its level had significantly risen after one year in PD group but not in controls. T-tau showed no significant change after 6 months and one year in control. T-tau levels had decreased significantly after 6 months in PD group, but no significant change was observed from baseline to visit 4 (after one year). Aβ_1−42_ had not significantly changed after 6 months, but it had significantly increased after one year in both control and PD groups.

**Figure 1:**
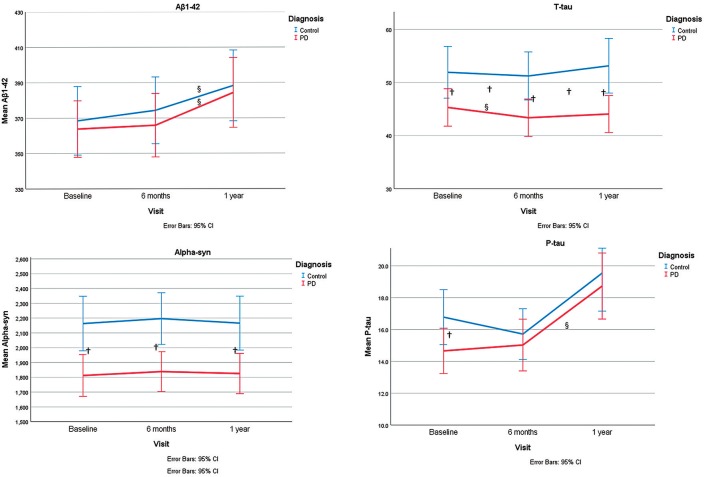
Representation of α-syn, P-tau, T-tau, and Aβ 1-42 levels at different time points and the trend of changes during study period. ^§^shows significant changes during time within each group within a linear mixed model. ^†^shows the significant group differences for CSF levels of markers at each time point or their changes (from baseline to 6 months or from baseline to 1 year).

On the whole, the pattern of changes (increase or decrease) of biomarkers was not different between controls and PD group, except for total tau for which there was a different pattern of change between the two groups. Also, the trend of changes based on interaction analysis of time and group, within the linear mixed model, was not significantly different between the two groups. (Table 2, Figure 1).

### Correlation between levels of different CSF biomarkers at different time points

None of the CSF biomarkers showed any significant correlation with each other in control group when adjusted for age and sex although several significant correlations were observed in control group when not adjusted. Correlations between levels of the same biomarker at different time points were significant in the PD group whether adjustment for age, gender, and disease duration was done or not. However, there were some exceptions for t-tau.

### Correlation of baseline values of each biomarker with estimated changes of different biomarkers during study period

**Figure 2:**
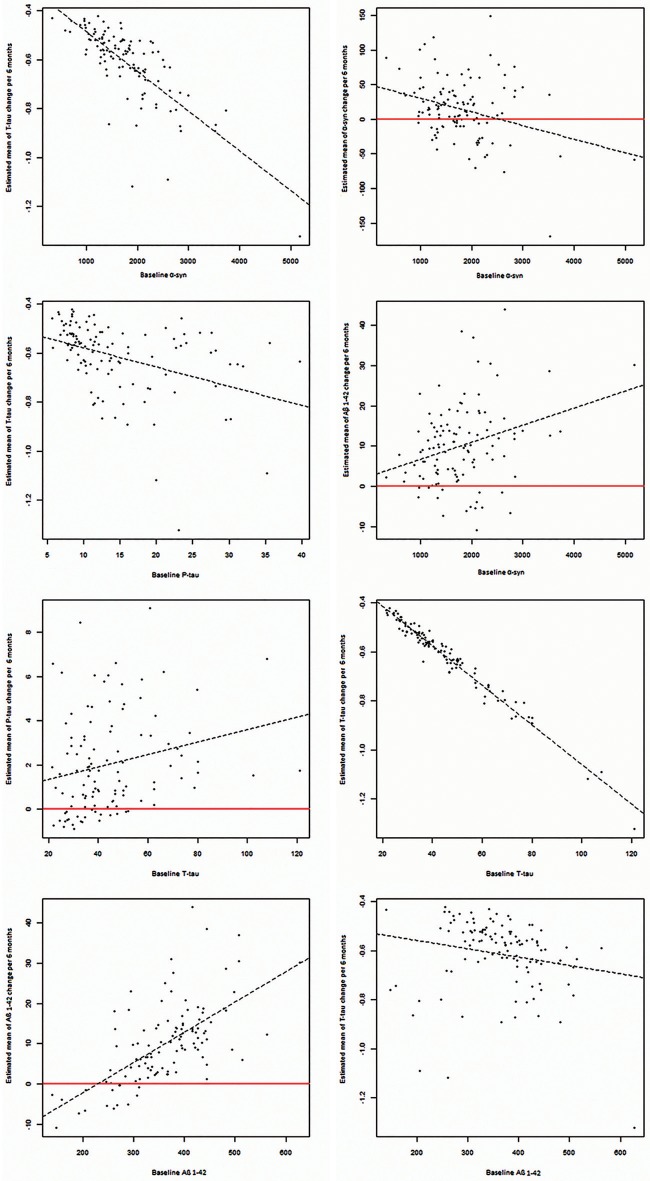
Significant correlations between baseline CSF levels of α-syn, P-tau, T-tau, and Aβ_1−42_ with estimated changes of α-syn, P-tau, T-tau, and Aβ_1−42_.

Baseline values of all biomarkers were correlated with their own estimated changes, except for p-tau. Additionally, baseline values of all biomarkers were correlated with estimated changes of t-tau and baseline values of α-syn correlated to estimated changes of Aβ_1−42_ (*P* = 0.002). (Table 3, Figure 2).

**Table 3:** The correlation and regression coefficient of estimated changes and baseline values with estimated change of different factors during study periods, obtained within linear mixed model.

**Estimated**	Estimated changes per year^*^				**Baseline values**				
Change per year^*^		**α-synuclein**	**P-tau**	**T-tau**	**Aβ42**	**α-synuclein**	**P-tau**	**T-tau**	**Aβ42**
α-synuclein	r		0.015	0.092	0.096	−0.310	−0.036	−0.125	0.042
	B		0.309	27.400	0.429	−0.020	−0.211	−0.306	0.022
	P		0.871	0.335	0.314	0.001	0.708	0.190	0.663
P-tau	r	0.015		−0.220	0.387	0.129	0.030	0.228	−0.068
	B	0.001		−3.286	0.087	0.000	0.009	0.028	−0.002
	P	0.871		0.020	0.000	0.175	0.750	0.016	0.476
T–tau	r	0.092	−0.220		−0.159	−0.762	−0.399	−0.983	−0.193
	B	0.000	−0.015		−0.002	0.000	−0.008	−0.008	0.000
	P	0.335	0.020		0.094	0.000	0.000	0.000	0.041
Aβ1-42	r	0.096	0.387	−0.159		0.297	−0.052	0.127	0.635
	B	0.021	1.731	−10.599		0.004	−0.068	0.070	0.075
	P	0.314	0.000	0.094		0.002	0.587	0.182	0.000

*r, Correlation coefficient*.

*B, Linear regression coefficient*.

* *Estimated changes were calculated for each subject based on the regression coefficient of each subject within a linear mixed model*.

*The correlations were assessed between different biomarkers in the study. One single biomarker could not have a correlationship with itself and they were marked black*.

### Correlation of estimated changes of different biomarkers during study period with each other

**Figure 3:**
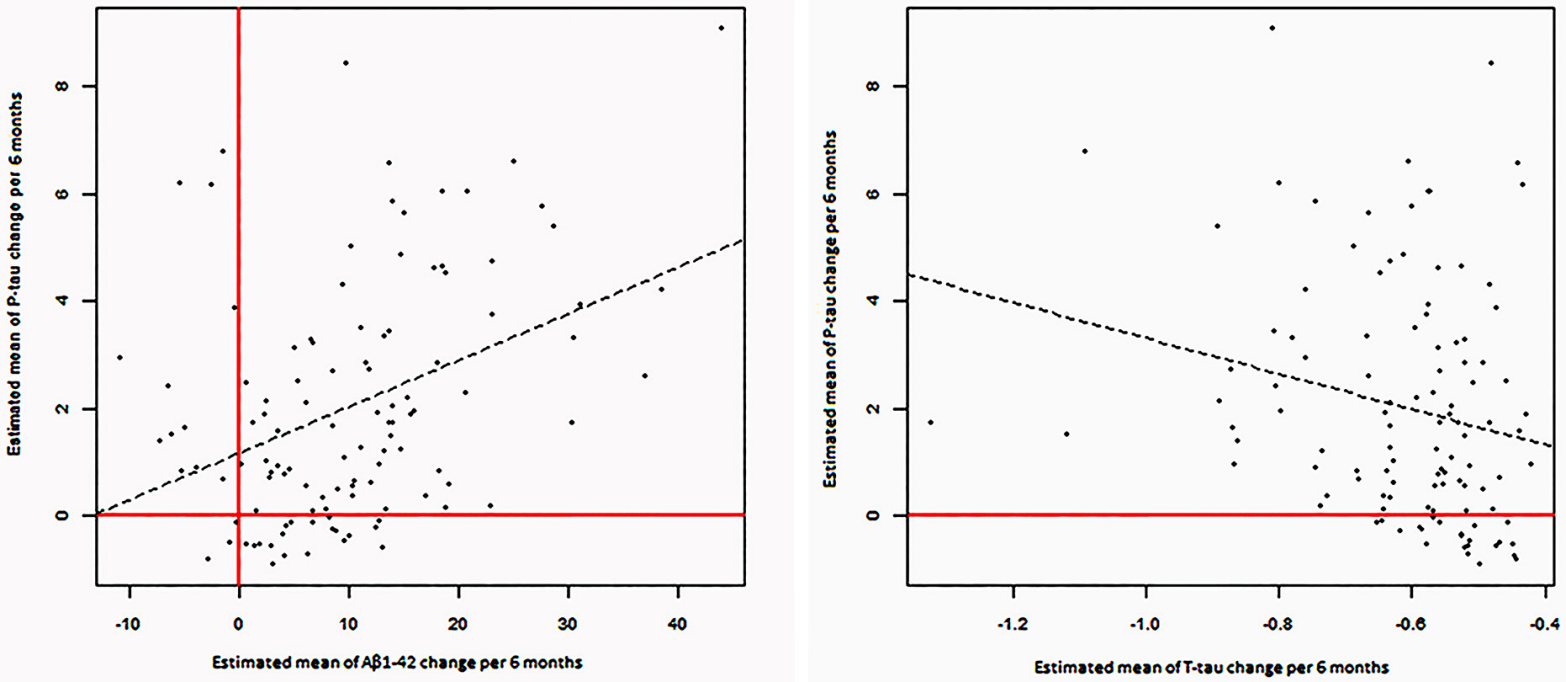
Significant correlations between estimated changes in CSF levels of α-syn, P-tau, T-tau, and Aβ 1- 42.

Estimated changes of p-tau and t-tau were positively correlated to each other (*P* = 0.002). Moreover, estimated changes of p-tau and Aβ_1−42_ were correlated positively (*P* < 0.001). (Table 3, Figure 3).

### Correlation of baseline values of MoCA, RBDSQ, UPSIT, and UPDRS III scores with estimated changes of different biomarkers during study

Using linear mixed models for changes of p-tau, t-tau, α-syn, and Aβ CSF levels, with interaction of baseline UPDRS III, RBDSQ, UPSIT, and MoCA scores, no significant association between these clinical scores with estimated changes of each CSF marker was observed except for the significant correlation between estimated changes in alpha-synuclein level and RBDSQ scores (*p* = 0.0013) (data not shown).

## Discussion

In this study, we explored baseline levels and longitudinal changes of CSF markers and evaluated their association with each other in drug-naïve, non-demented PD patients of PPMI cohort who were at the early stage of their disease. In addition, we evaluated the power of baseline clinical scores (UPDRS III, MoCA, UPSIT, RBDSQ) in prediction of longitudinal changes of CSF markers in PD patients. In brief, all CSF markers were lower in PDs compared to controls except Aβ_1−42_ at baseline, which was similar to the results of the previously mentioned cross-sectional study in PPMI cohort. In longitudinal assessment, Aβ_1−42_ and p-tau concentrations showed an increase in PD patients with the former also increasing in control subjects. Correlations between baseline concentrations of each of the CSF markers with their own estimated changes were significant except for p-tau. Furthermore, longitudinal changes of p-tau levels were positively correlated to t-tau and Aβ_1−42_ longitudinal changes.

### Difference between PD and control in biomarker levels at different time points and their changes during time

This study has shown a reduced level of α-syn and t-tau at all time-points and p-tau only at baseline in patients' CSF compared to healthy controls'. This observation is in line with the most recent studies (16, 20, 21); however, some studies observed higher or unchanged CSF t-tau or p-tau levels in PD patients (17, 29–31).

P-tau levels were significantly lower in the PD group compared to controls and rose significantly during the 1-year follow-up time, only in the PD group. Therefore, the p-tau level differences between PD and control groups vanished at the 6-month and one-year time points. On the other hand, t-tau levels were different between the two groups at all time points despite their non-significant longitudinal changes. In the study by Boungiorno et al., t-tau but not p-tau longitudinal increase was significant. Hall et al., however, found an increase in both. Thus, due to the high rate of p-tau changes during the disease course, at least in some time points, there would be an overlap with p-tau levels in controls, which challanges its diagnostic value. Thus, we hypothesize that CSF t-tau is a better candidate as a diagnostic tool than p-tau, which needs to be further investigated.

Our study does not provide the means to discover the underlying mechanism of these protein level differences; however, we speculate that at the very early stages of PD, i.e., at our baseline, there is an accumulation of toxic p-tau in form of neurofibrillary tangles (NFTs) in neurons, which causes absorption of more functional tau molecules from extracellular space as a compensatory mechanism in order to reverse back the function of neuronal trafficking. The similar mechanisms are the case for α-syn. In contrast, it has been shown that due to the altered processing of α-syn and occasionally increased transcription of SNCA gene, neurons secrete exosomal α-syn and propagate PD pathology (32, 33). Maybe, the observed changes of α-syn in different studies vary based on the weight of such mechanisms. This phenomenon causes a decrease in t-tau, p-tau, and α-syn levels in CSF. In later phases of the disease, due to axonal damage, p-tau and α-syn molecules accumulated in form of NFTs and Lewy bodies are released into CSF and the levels of p-tau and α-syn will increase. However, α-syn longitudinal changes are not significant in the time points we have studied, which is in line with some of the previous studies (26). One theory is that in PD the accelerated injury to neuronal plasma membrane which causes an increase in CSF α-syn, is contradicted by the more α-syn intracellular accumulation (34). Thus, variability in rates of axonal degeneration is one of the important factors in this regard and probably α-syn change is not an appropriate diagnostic tool for PD (35). We reckon that levels of t-tau and α-syn might have already fallen in earlier stages (because of absorption of these molecules into neurons as a compensation) and in the follow-up time due to the floor effect, no longitudinal decrease could be observed, although heterogeneity in disease duration makes such assumptions non-applicable. Maybe, longer follow-up times could allow significant longitudinal changes.

It is worthy of note that in AD, CSF tau levels are elevated from the earliest phases of the disease (36, 37) except for the subgroups of patients with the concomitant alpha-synucleinopathy, i.e., Dementia with Lewy Bodies (DLB) (37). This common observation in synucleinopathies might refer to the colocalization of tau molecules with α-syn, which contributes to their reduction in CSF.

On the other hand, Aβ_1−42_ levels were not significantly different between the two groups at any time point although significant longitudinal increase of Aβ_1−42_ was observed in both groups. However, the study done by Buongiorno et al. has shown the opposite, i.e., a decrease in Aβ levels during 18 months, which was associated with cognitive decline in non-demented PD patients (27). In addition, in the study done by Hall et al. the changes in Aβ levels in PD patients with long disease durations were non-significant during 2 years. Herein, we checked for levels of whole CSF Aβ (Aβ_1−42_) in contrast to the mentioned study, which evaluated the level of Aβ_42_, and this might explain the discrepancies in results. The progressive reduction of Aβ_1−42_ levels in other studies (both in AD and PD) can be mainly attributed to its extracellular accumulation but we can not justify the observed increase in Aβ_1−42_ in our study.

On the whole, it seems that the difference in clinical and pathophysiological characteristics contributes to variability in disease progression and different results of such studies.

Variables which affect the results of these studies include the rate of axonal damage, clinical endophenotypes, the stage of the disease, and whether compensatory mechanisms are working properly. For instance, it is shown that significantly increased levels of CSF α-syn and t-tau is a common phenomenon in the ones who develop PD dementia (PDD) (16). This is similar to the observation of increased α-syn and t-tau in AD patients compared to healthy controls (36–38). Herein, we excluded the patients fulfilling the criteria for PDD at baseline, and this might explain the reason why t-tau and α-syn did not increase during the course of the disease in our sample. Moreover, it seems that as the disease duration increases, higher levels of α-syn in patients compared to HC is encountered in CSF samples as opposed to the lower levels of this protein in early-stage PD cases such as ours. This is in line with the previous longitudinal assessment done by Hall et al. in which despite significant increases in all CSF markers except Aβ_42_, non-significantly changed level of CSF proteins in patients with short disease duration (<5 years from diagnosis) and controls were observed (21). Similarly, all patients in our study had short disease duration (<2 years) and also due to shorter follow-up time in our study, the time required for significant changes in some of the biomarkers was not provided.

### Correlation between levels of different CSF biomarkers at different time points and between their estimated changes

This study showed the correlation between different CSF markers adjusted for age and gender at different time points in PD patients. No significant correlation found in control group when adjusted for age and gender. In addition, lower baseline levels of p-tau, t-tau, and α-syn were correlated with lower rate of estimated decrease of t-tau within a linear mixed model.

The disappearance of any association between CSF biomarkers in controls after adjusting for age and gender made us infer that any observed correlation between protein concentrations in healthy people was confounded by age and gender. However, in PD group correlations remained significant after this adjustment; meaning that there must be a pathological and disease-specific link between these proteinopathies. Therefore, we suspect that any correlation observed between levels of different biomarkers in control cases of previous PPMI studies was the result of not adjusting them for age and gender (20).

Probably the most distinctive part of this study was the assessment of correlation between “estimated” changes of biomarkers within a linear mixed model and baseline values of different biomarkers. Lower p-tau, t-tau, and α-syn levels at baseline were predictive of the higher rate of t-tau level decrease in the next year. The power of lower α-syn levels at baseline for estimation of lower rate of *t*-tau decrease might be due to lower baseline α-syn CSF levels being associated with more toxic α-syn accumulation in Lewy bodies. Actually, accumulation of α-syn in Lewy body has a seeding effect on tau oligomerization and accumulation (11–13). In addition, accumulation of tau itself propagates its accumulation in neurofibrillary tangles. These protein accumulations (α-syn and tau) inhibit release of tau into CSF (39). Although, these phenomena necessitate more tau absorption into neurons as a compensatory mechanism, in later stages such mechanisms do not seem to function and there is lower rate of decrease in tau levels (making these changes were not significant in a one-year follow-up due to the floor effect or short follow-up time).

On the other hand, lower levels of Aβ_1−42_ were predictive of the lower rate of tau decrease in CSF. We suppose that lower level of CSF Aβ_1−42_ means more accumulation of Aβ in extracellular amyloid plaques and lead to lower free (intracellular) Aβ level, which is necessary for tau phosphorylation and accumulation (40).

On the whole, these results show that changes of t-tau CSF levels may be the convergence point of PD pathology. However, because amyloidopathy and synucleinopathy have opposing effects on the rate of CSF tau level changes, assessing CSF t-tau levels can not reflect the severity of underlying proteinopathies and thus, the rate of t-tau CSF level changes may be quite small and need a long time to change. Therefore, in short follow-up times, like in our study, its changes would not be significant.

Another finding was that lower CSF α-syn levels at baseline were predictive of slower Aβ_1−42_ increase after one year. One explanation is that α-syn accumulation propagates Aβ accumulations and its lower CSF concentrations. For instance, a recent study has shown that non-amyloid beta component of human alpha-synuclein oligomers induces the formation of new Aβ oligomers (41).

### Correlation between baseline clinical entities of PD patients and longitudinal changes of CSF markers

It has been suggested that Rapid Eye Movement (REM) sleep Behavior Disorder (RBD) can be a clue to early diagnosis of neurodegenerative diseases (42), specifically it can help with early detection of PD (19, 43, 44). Affliction of brainstem structures which control REM sleep with alpha-synuclein protein inclusions in early phases of PD seems to be the underlying mechanism responsible for RBD (45).

In this study, we detected significant increase in CSF levels of alpha-synuclein in PD-RBD patients. Although a previous cross-sectional study in PD patients have revealed no association between CSF alpha-synuclein levels and RBD (46), prior investigations have revealed that CSF Prion Proteins (PrPs) are significantly elevated in PD patients with RBD compared to PD patients without sleep disorders (47), which might be suggestive of accelerated neuronal degeneration in PD-RBD patients and thus, introduce RBD as potentially the most appropriate clinical predictive marker of neuronal degeneration and disease progression in PD, as shown previously (48).

Importantly, changes in other CSF protein levels were not associated with UPDRS III, MoCA, and UPSIT scores. This might refer to the fact that motor symptoms, cognitive impairment, and olfaction dysfunction are not appropriate predictors of progression of neuronal degeneration in PD patients. In this study we have not assessed progression of different PD symptoms in association with the baseline clinical scores and it is worthy of note that although these CSF biomarkers pattern of changes in patients with various clinical entities at baseline might elucidate the speed of neurodegeneration to some extent, it does not necessarily mean that clinical manifestations progress is in line with CSF marker changes. However, it might help identify the speed of neurodegeneration at a molecular level and design more targeted therapies.

## Conclusion

Although there are some positive points to this study like recruitment of drug-naïve patients, it suffers from some limitations like short follow-up time. To reach a more comprehensive conclusion, further longitudinal assessments regarding the predictive role of CSF proteins and their different species in patients at different stages of the disease and with longer follow-ups are suggested.

In addition, the patients in this study were quite heterogeneous in terms of disease duration which renders this study to some limitations; for instance, the patterns of CSF protein changes of different subgroups at different disease stages may be different.

In addition, in the manuscript we have assumed that lower CSF concentration of proteins is due to their accumulation in extracellular and intracellular spaces. This assumption might not be the case and several undiscovered mechanisms may play a role in determining the CSF concentrations of these proteins. There are some other limitations such as various degrees of CSF concentrations due to altered states of hydration and the position of proteins in a caudo-rostral column from the lumbar region to the cerebral origin. Moreover, clinical application of the difficult and invasive procedure of lumbar puncture to determine CSF markers as a way to follow-up the patients suffering from neurodegenerative diseases seems unlikely and such studies may just help enlighten the pathological mechanisms of proteinopathies.

In sum, it seems that α-syn, t-tau, p-tau, and Aβ play a role in PD pathology and have bidirectional interactions with each other, which mostly converge on tau pathology and lead to changes of CSF tau levels. Most noticeably, colocalization of α-syn and t-tau molecules in intracellular inclusions cause a reduction in CSF α-syn and t-tau levels, in contrast to AD without synucleinopathies which is associated with higher levels of CSF tau.

Importantly, among the clinical measures we applied, only RBD was predictive of α-syn increase during time. This might refer to the fact that evaluating patients for RBD at baseline is far more important than motor, olfaction, or cognitive assessment for prognostic purposes.

We suggest that different subtypes of PD patients progress heterogeneously. Thus, conducting similar studies in patient subtypes with various clinical symptoms and genetic, epigenetic, and environmental predispositions may be helpful.

In addition, most of the studies have shown the role of lower Aβ levels at baseline in predicting cognitive decline in the future (49). However, recently, predictive roles for tau levels in cognitive decline have been shown in other neurodegenerative diseases like Alzheimer's disease (50) and Huntington's disease (51). This observation in AD has been attributed to the later position of tau in the preclinical phase of the disease. To the best of our knowledge, there has been no evidence about the temporality of changes in tau and Aβ pathology in PD, i.e., whether CSF tau levels start to rise (or maybe fall) in later stages of the disease compared to Aβ or not. Thus, it seems that conducting longitudinal studies are necessary to discover whether changes in Aβ or tau is a more appropriate predictor of cognitive decline in the future.

## Ethics statement

All procedures performed here, including human participants were in accordance with the ethical standards of the institutional research committee and with the 1964 Helsinki declaration and its later amendments or comparable ethical standards.

## Informed consent

Informed consent was obtained from all individual participants included in the study.

## Author contributions

MD, MA, AA-G, SP, AK contributed to the conception and design of the study; MY, AA-G, MD, SP contributed to data collection and analysis; and MD, SP, AK, AA-G contributed to writing the manuscript.

### Conflict of interest statement

The authors declare that the research was conducted in the absence of any commercial or financial relationships that could be construed as a potential conflict of interest. The reviewer CH and handling Editor declared their shared affiliation.

## Abbreviations

CSF: cerebrospinal fluid
PD: Parkinson's disease
HC: healthy controls
α-syn: alpha-synuclein
Aβ: beta-amyloid
t-tau: total tau
p-tau: phosphorylated tau.
