# Supplementary material for: Longitudinal Alterations of Alpha-Synuclein, Amyloid Beta, Total, and Phosphorylated Tau in Cerebrospinal Fluid and Correlations Between Their Changes in Parkinson's Disease
Source: Front Neurol. 2018 Jul 11;9:560. doi: 10.3389/fneur.2018.00560 (PMC6052894; doi:10.3389/fneur.2018.00560)
Supplement: Supplementary file 1 [file Table_1.DOCX]

**Supplementary table 1. Correlation between CSF protein levels in control group at different time points without adjustment.**

| Time |  |  |  | Values in each time | | | |  | Baseline values | | | | Age | Disease |
| --- | --- | --- | --- | --- | --- | --- | --- | --- | --- | --- | --- | --- | --- | --- |
|  |  |  |  | α-synuclein | P-tau | T-tau | Aβ42 |  | α-synuclein | P-tau | T-tau | Aβ42 |  | Duration |
| Baseline | Values | α-synuclein | r |  | .439** | .801** | .334** |  |  | .439** | .801** | .334** | -0.101 | 0.053 |
|  |  |  | P |  | 0.000 | 0.000 | 0.000 |  |  | 0.000 | 0.000 | 0.000 | 0.133 | 0.577 |
|  |  |  |  |  |  |  |  |  |  |  |  |  |  |  |
|  |  | P-tau | r | .439** |  | .529** | .269** |  | .439** |  | .529** | .269** | 0.011 | -0.043 |
|  |  |  | P | 0.000 |  | 0.000 | 0.000 |  | 0.000 |  | 0.000 | 0.000 | 0.871 | 0.649 |
|  |  |  |  |  |  |  |  |  |  |  |  |  |  |  |
|  |  | T-tau | r | .801** | .529** |  | .343** |  | .801** | .529** |  | .343** | -0.086 | 0.043 |
|  |  |  | P | 0.000 | 0.000 |  | 0.000 |  | 0.000 | 0.000 |  | 0.000 | 0.200 | 0.654 |
|  |  |  |  |  |  |  |  |  |  |  |  |  |  |  |
|  |  | Aβ42 | r | .334** | .269** | .343** |  |  | .334** | .269** | .343** |  | -0.004 | 0.057 |
|  |  |  | P | 0.000 | 0.000 | 0.000 |  |  | 0.000 | 0.000 | 0.000 |  | 0.958 | 0.547 |
| 1 year | Values | α-synuclein | r |  | .316** | .758** | .351** |  | .820** | .415** | .695** | .355** | -0.062 | 0.049 |
|  |  |  | P |  | 0.000 | 0.000 | 0.000 |  | 0.000 | 0.000 | 0.000 | 0.000 | 0.356 | 0.609 |
|  |  |  |  |  |  |  |  |  |  |  |  |  |  |  |
|  |  | P-tau | r | .316** | 1.000 | .496** | .233** |  | .363** | .423** | .499** | .185** | 0.026 | 0.123 |
|  |  |  | P | 0.000 | 0.000 | 0.000 | 0.000 |  | 0.000 | 0.000 | 0.000 | 0.006 | 0.699 | 0.198 |
|  |  |  |  |  |  |  |  |  |  |  |  |  |  |  |
|  |  | T-tau | r | .758** | .496** | 1.000 | .318** |  | .768** | .497** | .907** | .299** | -0.038 | 0.084 |
|  |  |  | P | 0.000 | 0.000 | 0.000 | 0.000 |  | 0.000 | 0.000 | 0.000 | 0.000 | 0.576 | 0.378 |
|  |  |  |  |  |  |  |  |  |  |  |  |  |  |  |
|  |  | Aβ42 | r | .351** | .233** | .318** | 1.000 |  | .289** | .218** | .290** | .826** | 0.002 | -0.019 |
|  |  |  | P | 0.000 | 0.000 | 0.000 | 0.000 |  | 0.000 | 0.001 | 0.000 | 0.000 | 0.976 | 0.845 |
|  |  |  |  |  |  |  |  |  |  |  |  |  |  |  |
|  | Changes | α-synuclein change | r | .195** | -0.073 | -0.026 | 0.053 |  | -.297** | -0.011 | -0.132 | 0.013 | 0.107 | -0.121 |
|  |  |  | P | 0.003 | 0.280 | 0.703 | 0.433 |  | 0.000 | 0.869 | 0.050 | 0.848 | 0.112 | 0.206 |
|  |  |  |  |  |  |  |  |  |  |  |  |  |  |  |
|  |  | P-tau change | r | -0.066 | .466** | -0.002 | 0.034 |  | -0.047 | -.508** | -0.034 | -0.067 | -0.023 | 0.185 |
|  |  |  | P | 0.325 | 0.000 | 0.982 | 0.615 |  | 0.487 | 0.000 | 0.614 | 0.318 | 0.734 | 0.051 |
|  |  |  |  |  |  |  |  |  |  |  |  |  |  |  |
|  |  | T-tau change | r | -0.009 | -0.098 | 0.020 | 0.004 |  | -.166* | -.154* | -.327** | -.135* | 0.129 | -0.001 |
|  |  |  | P | 0.898 | 0.147 | 0.765 | 0.950 |  | 0.013 | 0.022 | 0.000 | 0.045 | 0.054 | 0.989 |
|  |  |  |  |  |  |  |  |  |  |  |  |  |  |  |
|  |  | Aβ42 change | r | 0.030 | 0.112 | 0.019 | .349** |  | -0.070 | -0.103 | -0.101 | -.184** | 0.025 | -0.118 |
|  |  |  | P | 0.652 | 0.097 | 0.774 | 0.000 |  | 0.303 | 0.125 | 0.132 | 0.006 | 0.709 | 0.214 |
|  |  |  |  |  |  |  |  |  |  |  |  |  |  |  |
|  | %Changes | %change in α-synuclein | r | .188** | -0.092 | -0.028 | 0.067 |  | -.308** | -0.007 | -0.130 | 0.032 | 0.095 | -0.128 |
|  |  |  | P | 0.005 | 0.173 | 0.680 | 0.322 |  | 0.000 | 0.922 | 0.054 | 0.640 | 0.160 | 0.180 |
|  |  |  |  |  |  |  |  |  |  |  |  |  |  |  |
|  |  | %change in P-tau | r | -0.066 | .465** | -0.009 | 0.016 |  | -0.053 | -.521** | -0.047 | -0.081 | -0.018 | 0.167 |
|  |  |  | P | 0.327 | 0.000 | 0.896 | 0.809 |  | 0.431 | 0.000 | 0.487 | 0.227 | 0.791 | 0.078 |
|  |  |  |  |  |  |  |  |  |  |  |  |  |  |  |
|  |  | %change in T-tau | r | 0.079 | -0.088 | 0.112 | 0.026 |  | -0.069 | -0.124 | -.243** | -0.108 | 0.108 | 0.011 |
|  |  |  | P | 0.240 | 0.192 | 0.097 | 0.698 |  | 0.307 | 0.065 | 0.000 | 0.110 | 0.110 | 0.907 |
|  |  |  |  |  |  |  |  |  |  |  |  |  |  |  |
|  |  | %change in Aβ42 | r | 0.019 | 0.117 | -0.001 | .342** |  | -0.089 | -0.112 | -0.116 | -.178** | 0.002 | -0.112 |
|  |  |  | P | 0.778 | 0.082 | 0.988 | 0.000 |  | 0.187 | 0.096 | 0.085 | 0.008 | 0.979 | 0.240 |
| 2 years | Values | α-synuclein | r |  | .189** | .758** | .324** |  | .800** | .382** | .719** | .349** | -0.105 | 0.122 |
|  |  |  | P |  | 0.005 | 0.000 | 0.000 |  | 0.000 | 0.000 | 0.000 | 0.000 | 0.120 | 0.199 |
|  |  |  |  |  |  |  |  |  |  |  |  |  |  |  |
|  |  | P-tau | r | .189** | 1.000 | .315** | .283** |  | .199** | .188** | .291** | -0.011 | 0.118 | 0.015 |
|  |  |  | P | 0.005 | 0.000 | 0.000 | 0.000 |  | 0.003 | 0.005 | 0.000 | 0.865 | 0.078 | 0.875 |
|  |  |  |  |  |  |  |  |  |  |  |  |  |  |  |
|  |  | T-tau | r | .758** | .315** | 1.000 | .311** |  | .752** | .515** | .905** | .306** | -0.081 | 0.028 |
|  |  |  | P | 0.000 | 0.000 | 0.000 | 0.000 |  | 0.000 | 0.000 | 0.000 | 0.000 | 0.229 | 0.768 |
|  |  |  |  |  |  |  |  |  |  |  |  |  |  |  |
|  |  | Aβ42 | r | .324** | .283** | .311** | 1.000 |  | .296** | .150* | .271** | .760** | -0.029 | -0.054 |
|  |  |  | P | 0.000 | 0.000 | 0.000 | 0.000 |  | 0.000 | 0.026 | 0.000 | 0.000 | 0.670 | 0.572 |
|  |  |  |  |  |  |  |  |  |  |  |  |  |  |  |
|  | Changes | α-synuclein change | r | .334** | 0.027 | 0.086 | 0.067 |  | -.209** | -0.035 | -0.047 | 0.034 | 0.050 | 0.076 |
|  |  |  | P | 0.000 | 0.689 | 0.199 | 0.321 |  | 0.002 | 0.606 | 0.487 | 0.618 | 0.458 | 0.427 |
|  |  |  |  |  |  |  |  |  |  |  |  |  |  |  |
|  |  | P-tau change | r | -0.064 | .707** | -0.059 | .165* |  | -0.102 | -.479** | -0.092 | -.164* | 0.072 | 0.065 |
|  |  |  | P | 0.340 | 0.000 | 0.378 | 0.014 |  | 0.130 | 0.000 | 0.170 | 0.015 | 0.283 | 0.496 |
|  |  |  |  |  |  |  |  |  |  |  |  |  |  |  |
|  |  | T-tau change | r | 0.033 | -0.007 | .137* | 0.063 |  | -0.114 | -0.084 | -.221** | -0.100 | -0.013 | -0.035 |
|  |  |  | P | 0.626 | 0.921 | 0.041 | 0.347 |  | 0.090 | 0.215 | 0.001 | 0.136 | 0.846 | 0.718 |
|  |  |  |  |  |  |  |  |  |  |  |  |  |  |  |
|  |  | Aβ42 change | r | 0.037 | .454** | 0.077 | .473** |  | -0.001 | -.148* | -0.044 | -.150* | -0.032 | -0.167 |
|  |  |  | P | 0.584 | 0.000 | 0.254 | 0.000 |  | 0.989 | 0.028 | 0.514 | 0.025 | 0.636 | 0.078 |
|  |  |  |  |  |  |  |  |  |  |  |  |  |  |  |
|  | %Changes | %change in α-synuclein | r | .330** | 0.017 | 0.074 | 0.064 |  | -.226** | -0.042 | -0.059 | 0.048 | 0.038 | 0.069 |
|  |  |  | P | 0.000 | 0.799 | 0.270 | 0.339 |  | 0.001 | 0.532 | 0.385 | 0.476 | 0.570 | 0.471 |
|  |  |  |  |  |  |  |  |  |  |  |  |  |  |  |
|  |  | %change in P-tau | r | -0.115 | .682** | -0.101 | .136* |  | -.168* | -.515** | -.137* | -.182** | 0.066 | 0.048 |
|  |  |  | P | 0.087 | 0.000 | 0.133 | 0.043 |  | 0.012 | 0.000 | 0.042 | 0.007 | 0.324 | 0.615 |
|  |  |  |  |  |  |  |  |  |  |  |  |  |  |  |
|  |  | %change in T-tau | r | 0.037 | -0.005 | .134* | 0.055 |  | -0.115 | -0.084 | -.231** | -0.102 | -0.003 | -0.027 |
|  |  |  | P | 0.583 | 0.941 | 0.047 | 0.415 |  | 0.088 | 0.211 | 0.001 | 0.131 | 0.967 | 0.777 |
|  |  |  |  |  |  |  |  |  |  |  |  |  |  |  |
|  |  | %change in Aβ42 | r | -0.006 | .422** | 0.031 | .440** |  | -0.043 | -.175** | -0.087 | -.169* | -0.022 | -0.180 |
|  |  |  | P | 0.926 | 0.000 | 0.641 | 0.000 |  | 0.523 | 0.009 | 0.197 | 0.012 | 0.746 | 0.058 |

r: Spearman correlation coefficient.

* P-values < 0.05.

** P-values < 0.001.

**Supplementary table 2. Correlation between CSF protein levels in PD group at different time points without adjustment.**

| Time |  |  |  | Values in each time | | | |  | Baseline values | | | | Age | Disease |
| --- | --- | --- | --- | --- | --- | --- | --- | --- | --- | --- | --- | --- | --- | --- |
|  |  |  |  | α-synuclein | P-tau | T-tau | Aβ42 |  | α-synuclein | P-tau | T-tau | Aβ42 |  | Duration |
| Baseline | Values | α-synuclein | r |  | .388** | .742** | .323** |  |  | .388** | .742** | .323** | -0.104 | 0.053 |
|  |  |  | P |  | 0.000 | 0.000 | 0.001 |  |  | 0.000 | 0.000 | 0.001 | 0.274 | 0.577 |
|  |  |  |  |  |  |  |  |  |  |  |  |  |  |  |
|  |  | P-tau | r | .388** |  | .514** | .250** |  | .388** |  | .514** | .250** | 0.026 | -0.043 |
|  |  |  | P | 0.000 |  | 0.000 | 0.008 |  | 0.000 |  | 0.000 | 0.008 | 0.787 | 0.649 |
|  |  |  |  |  |  |  |  |  |  |  |  |  |  |  |
|  |  | T-tau | r | .742** | .514** |  | .335** |  | .742** | .514** |  | .335** | -0.035 | 0.043 |
|  |  |  | P | 0.000 | 0.000 |  | 0.000 |  | 0.000 | 0.000 |  | 0.000 | 0.713 | 0.654 |
|  |  |  |  |  |  |  |  |  |  |  |  |  |  |  |
|  |  | Aβ42 | r | .323** | .250** | .335** |  |  | .323** | .250** | .335** |  | 0.037 | 0.057 |
|  |  |  | P | 0.001 | 0.008 | 0.000 |  |  | 0.001 | 0.008 | 0.000 |  | 0.702 | 0.547 |
| 1 year | Values | α-synuclein | r |  | .199* | .744** | .326** |  | .862** | .393** | .712** | .380** | -0.059 | 0.049 |
|  |  |  | P |  | 0.035 | 0.000 | 0.000 |  | 0.000 | 0.000 | 0.000 | 0.000 | 0.536 | 0.609 |
|  |  |  |  |  |  |  |  |  |  |  |  |  |  |  |
|  |  | P-tau | r | .199* | 1.000 | .387** | .259** |  | .203* | .294** | .382** | 0.184 | 0.075 | 0.123 |
|  |  |  | P | 0.035 | 0.000 | 0.000 | 0.006 |  | 0.032 | 0.002 | 0.000 | 0.052 | 0.430 | 0.198 |
|  |  |  |  |  |  |  |  |  |  |  |  |  |  |  |
|  |  | T-tau | r | .744** | .387** | 1.000 | .276** |  | .749** | .461** | .910** | .286** | 0.019 | 0.084 |
|  |  |  | P | 0.000 | 0.000 | 0.000 | 0.003 |  | 0.000 | 0.000 | 0.000 | 0.002 | 0.841 | 0.378 |
|  |  |  |  |  |  |  |  |  |  |  |  |  |  |  |
|  |  | Aβ42 | r | .326** | .259** | .276** | 1.000 |  | .252** | 0.163 | .273** | .815** | 0.076 | -0.019 |
|  |  |  | P | 0.000 | 0.006 | 0.003 | 0.000 |  | 0.007 | 0.086 | 0.004 | 0.000 | 0.427 | 0.845 |
|  |  |  |  |  |  |  |  |  |  |  |  |  |  |  |
|  | Changes | α-synuclein change | r | .225* | -0.020 | 0.006 | 0.121 |  | -.224* | 0.024 | -0.021 | 0.109 | 0.094 | -0.121 |
|  |  |  | P | 0.017 | 0.832 | 0.946 | 0.203 |  | 0.017 | 0.802 | 0.823 | 0.252 | 0.323 | 0.206 |
|  |  |  |  |  |  |  |  |  |  |  |  |  |  |  |
|  |  | P-tau change | r | -0.103 | .554** | -0.023 | 0.184 |  | -0.102 | -.526** | -0.110 | 0.031 | 0.001 | 0.185 |
|  |  |  | P | 0.281 | 0.000 | 0.809 | 0.052 |  | 0.283 | 0.000 | 0.250 | 0.745 | 0.988 | 0.051 |
|  |  |  |  |  |  |  |  |  |  |  |  |  |  |  |
|  |  | T-tau change | r | -0.002 | -0.077 | 0.033 | -0.032 |  | -0.061 | -.200* | -.304** | -0.148 | 0.134 | -0.001 |
|  |  |  | P | 0.981 | 0.420 | 0.727 | 0.741 |  | 0.521 | 0.035 | 0.001 | 0.120 | 0.160 | 0.989 |
|  |  |  |  |  |  |  |  |  |  |  |  |  |  |  |
|  |  | Aβ42 change | r | 0.015 | .207* | 0.037 | .449** |  | -0.043 | -0.097 | -0.055 | -0.092 | 0.076 | -0.118 |
|  |  |  | P | 0.871 | 0.029 | 0.701 | 0.000 |  | 0.651 | 0.310 | 0.563 | 0.332 | 0.424 | 0.214 |
|  |  |  |  |  |  |  |  |  |  |  |  |  |  |  |
|  | %Changes | %change in α-synuclein | r | .196* | -0.048 | -0.021 | 0.108 |  | -.255** | 0.025 | -0.040 | 0.100 | 0.084 | -0.128 |
|  |  |  | P | 0.039 | 0.615 | 0.822 | 0.258 |  | 0.007 | 0.793 | 0.678 | 0.296 | 0.376 | 0.180 |
|  |  |  |  |  |  |  |  |  |  |  |  |  |  |  |
|  |  | %change in P-tau | r | -0.135 | .545** | -0.048 | 0.162 |  | -0.126 | -.547** | -0.135 | 0.006 | 0.002 | 0.167 |
|  |  |  | P | 0.156 | 0.000 | 0.612 | 0.088 |  | 0.187 | 0.000 | 0.155 | 0.949 | 0.986 | 0.078 |
|  |  |  |  |  |  |  |  |  |  |  |  |  |  |  |
|  |  | %change in T-tau | r | 0.128 | -0.079 | 0.153 | -0.002 |  | 0.082 | -0.151 | -.193* | -0.100 | 0.098 | 0.011 |
|  |  |  | P | 0.177 | 0.405 | 0.106 | 0.980 |  | 0.388 | 0.111 | 0.041 | 0.295 | 0.303 | 0.907 |
|  |  |  |  |  |  |  |  |  |  |  |  |  |  |  |
|  |  | %change in Aβ42 | r | -0.007 | .224* | 0.018 | .429** |  | -0.071 | -0.100 | -0.070 | -0.103 | 0.041 | -0.112 |
|  |  |  | P | 0.943 | 0.017 | 0.850 | 0.000 |  | 0.455 | 0.292 | 0.460 | 0.279 | 0.668 | 0.240 |
| 2 years | Values | α-synuclein | r |  | 0.055 | .681** | .289** |  | .805** | .349** | .665** | .342** | -0.056 | 0.122 |
|  |  |  | P |  | 0.566 | 0.000 | 0.002 |  | 0.000 | 0.000 | 0.000 | 0.000 | 0.556 | 0.199 |
|  |  |  |  |  |  |  |  |  |  |  |  |  |  |  |
|  |  | P-tau | r | 0.055 | 1.000 | .252** | .283** |  | 0.091 | 0.134 | .247** | -0.074 | 0.112 | 0.015 |
|  |  |  | P | 0.566 | 0.000 | 0.007 | 0.003 |  | 0.340 | 0.159 | 0.009 | 0.436 | 0.240 | 0.875 |
|  |  |  |  |  |  |  |  |  |  |  |  |  |  |  |
|  |  | T-tau | r | .681** | .252** | 1.000 | .312** |  | .716** | .506** | .923** | .332** | -0.015 | 0.028 |
|  |  |  | P | 0.000 | 0.007 | 0.000 | 0.001 |  | 0.000 | 0.000 | 0.000 | 0.000 | 0.871 | 0.768 |
|  |  |  |  |  |  |  |  |  |  |  |  |  |  |  |
|  |  | Aβ42 | r | .289** | .283** | .312** | 1.000 |  | .289** | 0.127 | .290** | .740** | -0.020 | -0.054 |
|  |  |  | P | 0.002 | 0.003 | 0.001 | 0.000 |  | 0.002 | 0.183 | 0.002 | 0.000 | 0.835 | 0.572 |
|  |  |  |  |  |  |  |  |  |  |  |  |  |  |  |
|  | Changes | α-synuclein change | r | .354** | 0.028 | 0.076 | 0.063 |  | -0.174 | -0.025 | -0.010 | 0.102 | 0.045 | 0.076 |
|  |  |  | P | 0.000 | 0.771 | 0.428 | 0.511 |  | 0.066 | 0.795 | 0.914 | 0.282 | 0.635 | 0.427 |
|  |  |  |  |  |  |  |  |  |  |  |  |  |  |  |
|  |  | P-tau change | r | -0.096 | .763** | -0.056 | .245** |  | -0.098 | -.450** | -0.064 | -0.142 | 0.054 | 0.065 |
|  |  |  | P | 0.314 | 0.000 | 0.555 | 0.009 |  | 0.303 | 0.000 | 0.505 | 0.137 | 0.574 | 0.496 |
|  |  |  |  |  |  |  |  |  |  |  |  |  |  |  |
|  |  | T-tau change | r | -0.010 | -0.034 | 0.111 | 0.129 |  | -0.100 | -0.073 | -.236* | 0.063 | 0.022 | -0.035 |
|  |  |  | P | 0.920 | 0.721 | 0.244 | 0.175 |  | 0.294 | 0.446 | 0.012 | 0.511 | 0.816 | 0.718 |
|  |  |  |  |  |  |  |  |  |  |  |  |  |  |  |
|  |  | Aβ42 change | r | 0.065 | .517** | 0.089 | .545** |  | 0.049 | -0.134 | 0.023 | -0.069 | -0.055 | -0.167 |
|  |  |  | P | 0.499 | 0.000 | 0.353 | 0.000 |  | 0.611 | 0.160 | 0.808 | 0.470 | 0.561 | 0.078 |
|  |  |  |  |  |  |  |  |  |  |  |  |  |  |  |
|  | %Changes | %change in α-synuclein | r | .330** | -0.006 | 0.051 | 0.038 |  | -.205* | -0.039 | -0.039 | 0.090 | 0.066 | 0.069 |
|  |  |  | P | 0.000 | 0.948 | 0.596 | 0.689 |  | 0.030 | 0.687 | 0.682 | 0.346 | 0.490 | 0.471 |
|  |  |  |  |  |  |  |  |  |  |  |  |  |  |  |
|  |  | %change in P-tau | r | -0.174 | .733** | -0.115 | .200* |  | -.190* | -.498** | -0.128 | -0.175 | 0.061 | 0.048 |
|  |  |  | P | 0.067 | 0.000 | 0.227 | 0.035 |  | 0.045 | 0.000 | 0.179 | 0.064 | 0.523 | 0.615 |
|  |  |  |  |  |  |  |  |  |  |  |  |  |  |  |
|  |  | %change in T-tau | r | 0.031 | -0.030 | 0.137 | 0.130 |  | -0.061 | -0.056 | -.216* | 0.077 | 0.004 | -0.027 |
|  |  |  | P | 0.745 | 0.753 | 0.151 | 0.171 |  | 0.524 | 0.556 | 0.022 | 0.419 | 0.964 | 0.777 |
|  |  |  |  |  |  |  |  |  |  |  |  |  |  |  |
|  |  | %change in Aβ42 | r | 0.059 | .494** | 0.082 | .509** |  | 0.035 | -0.154 | 0.016 | -0.098 | -0.040 | -0.180 |
|  |  |  | P | 0.535 | 0.000 | 0.393 | 0.000 |  | 0.711 | 0.105 | 0.870 | 0.302 | 0.674 | 0.058 |

r: Spearman correlation coefficient.

* P-values < 0.05.

** P-values < 0.001.

**Supplementary table 3. Correlation between CSF protein levels in control group at different time points with adjustment for age and sex.**

| Time |  |  |  | Values in each time | | | |  | Baseline values | | | |
| --- | --- | --- | --- | --- | --- | --- | --- | --- | --- | --- | --- | --- |
|  |  |  |  | α-synuclein | P-tau | T-tau | Aβ42 |  | α-synuclein | P-tau | T-tau | Aβ42 |
| Baseline | Values | α-synuclein | r |  | 0.357 | 0.784 | 0.245 |  |  | 0.357 | 0.784 | 0.245 |
|  |  |  | P |  | 0.000 | 0.000 | 0.011 |  |  | 0.000 | 0.000 | 0.011 |
|  |  |  |  |  |  |  |  |  |  |  |  |  |
|  |  | P-tau | r | 0.357 |  | 0.497 | 0.171 |  | 0.357 |  | 0.497 | 0.171 |
|  |  |  | P | 0.000 |  | 0.000 | 0.076 |  | 0.000 |  | 0.000 | 0.076 |
|  |  |  |  |  |  |  |  |  |  |  |  |  |
|  |  | T-tau | r | 0.784 | 0.497 |  | 0.073 |  | 0.784 | 0.497 |  | 0.073 |
|  |  |  | P | 0.000 | 0.000 |  | 0.455 |  | 0.000 | 0.000 |  | 0.455 |
|  |  |  |  |  |  |  |  |  |  |  |  |  |
|  |  | Aβ42 | r | 0.245 | 0.171 | 0.073 |  |  | 0.245 | 0.171 | 0.073 |  |
|  |  |  | P | 0.011 | 0.076 | 0.455 |  |  | 0.011 | 0.076 | 0.455 |  |
| 1 year | Values | α-synuclein | r |  | 0.400 | 0.734 | 0.204 |  | 0.776 | 0.385 | 0.682 | 0.160 |
|  |  |  | P |  | 0.000 | 0.000 | 0.035 |  | 0.000 | 0.000 | 0.000 | 0.097 |
|  |  |  |  |  |  |  |  |  |  |  |  |  |
|  |  | P-tau | r | 0.400 | 1.000 | 0.599 | 0.095 |  | 0.449 | 0.441 | 0.600 | 0.034 |
|  |  |  | P | 0.000 | 0.000 | 0.000 | 0.329 |  | 0.000 | 0.000 | 0.000 | 0.724 |
|  |  |  |  |  |  |  |  |  |  |  |  |  |
|  |  | T-tau | r | 0.734 | 0.599 | 1.000 | 0.073 |  | 0.726 | 0.502 | 0.941 | 0.049 |
|  |  |  | P | 0.000 | 0.000 | 0.000 | 0.453 |  | 0.000 | 0.000 | 0.000 | 0.617 |
|  |  |  |  |  |  |  |  |  |  |  |  |  |
|  |  | Aβ42 | r | 0.204 | 0.095 | 0.073 | 1.000 |  | 0.228 | 0.103 | 0.058 | 0.860 |
|  |  |  | P | 0.035 | 0.329 | 0.453 | 0.000 |  | 0.018 | 0.288 | 0.551 | 0.000 |
|  |  |  |  |  |  |  |  |  |  |  |  |  |
|  | Changes | α-synuclein change | r | 0.259 | -0.108 | -0.049 | -0.054 |  | -0.409 | 0.011 | -0.213 | -0.143 |
|  |  |  | P | 0.007 | 0.266 | 0.611 | 0.577 |  | 0.000 | 0.914 | 0.027 | 0.139 |
|  |  |  |  |  |  |  |  |  |  |  |  |  |
|  |  | P-tau change | r | -0.017 | 0.470 | 0.048 | -0.016 |  | 0.054 | -0.585 | 0.053 | -0.138 |
|  |  |  | P | 0.858 | 0.000 | 0.624 | 0.871 |  | 0.578 | 0.000 | 0.587 | 0.156 |
|  |  |  |  |  |  |  |  |  |  |  |  |  |
|  |  | T-tau change | r | 0.012 | -0.118 | -0.019 | 0.030 |  | -0.310 | -0.083 | -0.357 | -0.080 |
|  |  |  | P | 0.905 | 0.225 | 0.846 | 0.757 |  | 0.001 | 0.391 | 0.000 | 0.411 |
|  |  |  |  |  |  |  |  |  |  |  |  |  |
|  |  | Aβ42 change | r | 0.070 | 0.110 | 0.042 | 0.206 |  | -0.048 | -0.137 | -0.032 | -0.323 |
|  |  |  | P | 0.472 | 0.257 | 0.666 | 0.033 |  | 0.625 | 0.157 | 0.745 | 0.001 |
|  |  |  |  |  |  |  |  |  |  |  |  |  |
|  | %Changes | %change in α-synuclein | r | 0.191 | -0.118 | -0.129 | -0.092 |  | -0.380 | -0.053 | -0.247 | -0.168 |
|  |  |  | P | 0.047 | 0.223 | 0.182 | 0.345 |  | 0.000 | 0.589 | 0.010 | 0.083 |
|  |  |  |  |  |  |  |  |  |  |  |  |  |
|  |  | %change in P-tau | r | 0.064 | 0.423 | 0.090 | -0.057 |  | 0.117 | -0.528 | 0.085 | -0.175 |
|  |  |  | P | 0.509 | 0.000 | 0.356 | 0.561 |  | 0.228 | 0.000 | 0.382 | 0.071 |
|  |  |  |  |  |  |  |  |  |  |  |  |  |
|  |  | %change in T-tau | r | 0.058 | -0.097 | 0.062 | 0.067 |  | -0.224 | -0.088 | -0.256 | -0.046 |
|  |  |  | P | 0.549 | 0.318 | 0.526 | 0.493 |  | 0.020 | 0.366 | 0.008 | 0.635 |
|  |  |  |  |  |  |  |  |  |  |  |  |  |
|  |  | %change in Aβ42 | r | 0.068 | 0.045 | -0.001 | 0.180 |  | -0.044 | -0.149 | -0.067 | -0.285 |
|  |  |  | P | 0.483 | 0.645 | 0.991 | 0.063 |  | 0.650 | 0.124 | 0.493 | 0.003 |
| 2 years | Values | α-synuclein | r |  | 0.191 | 0.770 | 0.226 |  | 0.807 | 0.341 | 0.771 | 0.246 |
|  |  |  | P |  | 0.047 | 0.000 | 0.018 |  | 0.000 | 0.000 | 0.000 | 0.010 |
|  |  |  |  |  |  |  |  |  |  |  |  |  |
|  |  | P-tau | r | 0.191 | 1.000 | 0.329 | 0.080 |  | 0.208 | 0.110 | 0.327 | -0.133 |
|  |  |  | P | 0.047 | 0.000 | 0.000 | 0.410 |  | 0.031 | 0.258 | 0.001 | 0.170 |
|  |  |  |  |  |  |  |  |  |  |  |  |  |
|  |  | T-tau | r | 0.770 | 0.329 | 1.000 | 0.020 |  | 0.690 | 0.485 | 0.932 | 0.017 |
|  |  |  | P | 0.000 | 0.000 | 0.000 | 0.834 |  | 0.000 | 0.000 | 0.000 | 0.862 |
|  |  |  |  |  |  |  |  |  |  |  |  |  |
|  |  | Aβ42 | r | 0.226 | 0.080 | 0.020 | 1.000 |  | 0.185 | 0.034 | -0.002 | 0.810 |
|  |  |  | P | 0.018 | 0.410 | 0.834 | 0.000 |  | 0.055 | 0.729 | 0.985 | 0.000 |
|  |  |  |  |  |  |  |  |  |  |  |  |  |
|  | Changes | α-synuclein change | r | 0.301 | -0.029 | 0.121 | 0.064 |  | -0.320 | -0.030 | -0.028 | -0.002 |
|  |  |  | P | 0.002 | 0.770 | 0.211 | 0.512 |  | 0.001 | 0.757 | 0.773 | 0.985 |
|  |  |  |  |  |  |  |  |  |  |  |  |  |
|  |  | P-tau change | r | -0.049 | 0.785 | -0.021 | 0.047 |  | -0.045 | -0.530 | -0.031 | -0.220 |
|  |  |  | P | 0.615 | 0.000 | 0.827 | 0.627 |  | 0.642 | 0.000 | 0.751 | 0.022 |
|  |  |  |  |  |  |  |  |  |  |  |  |  |
|  |  | T-tau change | r | 0.098 | 0.050 | 0.309 | 0.061 |  | -0.156 | 0.032 | -0.057 | -0.144 |
|  |  |  | P | 0.315 | 0.609 | 0.001 | 0.530 |  | 0.107 | 0.743 | 0.561 | 0.136 |
|  |  |  |  |  |  |  |  |  |  |  |  |  |
|  |  | Aβ42 change | r | -0.021 | 0.344 | 0.006 | 0.346 |  | -0.088 | -0.219 | -0.119 | -0.269 |
|  |  |  | P | 0.829 | 0.000 | 0.947 | 0.000 |  | 0.367 | 0.023 | 0.219 | 0.005 |
|  |  |  |  |  |  |  |  |  |  |  |  |  |
|  | %Changes | %change in α-synuclein | r | 0.178 | -0.029 | 0.018 | 0.057 |  | -0.346 | -0.071 | -0.101 | 0.012 |
|  |  |  | P | 0.065 | 0.764 | 0.852 | 0.559 |  | 0.000 | 0.466 | 0.298 | 0.901 |
|  |  |  |  |  |  |  |  |  |  |  |  |  |
|  |  | %change in P-tau | r | -0.048 | 0.744 | -0.022 | 0.045 |  | -0.075 | -0.458 | -0.051 | -0.217 |
|  |  |  | P | 0.623 | 0.000 | 0.822 | 0.645 |  | 0.442 | 0.000 | 0.597 | 0.024 |
|  |  |  |  |  |  |  |  |  |  |  |  |  |
|  |  | %change in T-tau | r | 0.034 | 0.053 | 0.194 | 0.094 |  | -0.209 | -0.042 | -0.143 | -0.127 |
|  |  |  | P | 0.725 | 0.589 | 0.045 | 0.334 |  | 0.030 | 0.668 | 0.140 | 0.190 |
|  |  |  |  |  |  |  |  |  |  |  |  |  |
|  |  | %change in Aβ42 | r | -0.086 | 0.330 | -0.047 | 0.326 |  | -0.164 | -0.202 | -0.171 | -0.242 |
|  |  |  | P | 0.378 | 0.000 | 0.633 | 0.001 |  | 0.090 | 0.036 | 0.077 | 0.011 |

r: Partial correlation coefficient, adjusted for age and sex.

**Supplementary table 4. Correlation between CSF protein levels in PD group at different time points with adjustment for age, sex, and disease duration.**

| Time |  |  |  | Values in each time | | | |  | Baseline values | | | |
| --- | --- | --- | --- | --- | --- | --- | --- | --- | --- | --- | --- | --- |
|  |  |  |  | α-synuclein | P-tau | T-tau | Aβ42 |  | α-synuclein | P-tau | T-tau | Aβ42 |
| Baseline | Values | α-synuclein | r |  | .388** | .742** | .323** |  |  | .388** | .742** | .323** |
|  |  |  | P |  | 0.000 | 0.000 | 0.001 |  |  | 0.000 | 0.000 | 0.001 |
|  |  |  |  |  |  |  |  |  |  |  |  |  |
|  |  | P-tau | r | .388** |  | .514** | .250** |  | .388** |  | .514** | .250** |
|  |  |  | P | 0.000 |  | 0.000 | 0.008 |  | 0.000 |  | 0.000 | 0.008 |
|  |  |  |  |  |  |  |  |  |  |  |  |  |
|  |  | T-tau | r | .742** | .514** |  | .335** |  | .742** | .514** |  | .335** |
|  |  |  | P | 0.000 | 0.000 |  | 0.000 |  | 0.000 | 0.000 |  | 0.000 |
|  |  |  |  |  |  |  |  |  |  |  |  |  |
|  |  | Aβ42 | r | .323** | .250** | .335** |  |  | .323** | .250** | .335** |  |
|  |  |  | P | 0.001 | 0.008 | 0.000 |  |  | 0.001 | 0.008 | 0.000 |  |
| 1 year | Values | α-synuclein | r |  | .199* | .744** | .326** |  | .862** | .393** | .712** | .380** |
|  |  |  | P |  | 0.035 | 0.000 | 0.000 |  | 0.000 | 0.000 | 0.000 | 0.000 |
|  |  |  |  |  |  |  |  |  |  |  |  |  |
|  |  | P-tau | r | .199* | 1.000 | .387** | .259** |  | .203* | .294** | .382** | 0.184 |
|  |  |  | P | 0.035 | 0.000 | 0.000 | 0.006 |  | 0.032 | 0.002 | 0.000 | 0.052 |
|  |  |  |  |  |  |  |  |  |  |  |  |  |
|  |  | T-tau | r | .744** | .387** | 1.000 | .276** |  | .749** | .461** | .910** | .286** |
|  |  |  | P | 0.000 | 0.000 | 0.000 | 0.003 |  | 0.000 | 0.000 | 0.000 | 0.002 |
|  |  |  |  |  |  |  |  |  |  |  |  |  |
|  |  | Aβ42 | r | .326** | .259** | .276** | 1.000 |  | .252** | 0.163 | .273** | .815** |
|  |  |  | P | 0.000 | 0.006 | 0.003 | 0.000 |  | 0.007 | 0.086 | 0.004 | 0.000 |
|  |  |  |  |  |  |  |  |  |  |  |  |  |
|  | Changes | α-synuclein change | r | .225* | -0.020 | 0.006 | 0.121 |  | -.224* | 0.024 | -0.021 | 0.109 |
|  |  |  | P | 0.017 | 0.832 | 0.946 | 0.203 |  | 0.017 | 0.802 | 0.823 | 0.252 |
|  |  |  |  |  |  |  |  |  |  |  |  |  |
|  |  | P-tau change | r | -0.103 | .554** | -0.023 | 0.184 |  | -0.102 | -.526** | -0.110 | 0.031 |
|  |  |  | P | 0.281 | 0.000 | 0.809 | 0.052 |  | 0.283 | 0.000 | 0.250 | 0.745 |
|  |  |  |  |  |  |  |  |  |  |  |  |  |
|  |  | T-tau change | r | -0.002 | -0.077 | 0.033 | -0.032 |  | -0.061 | -.200* | -.304** | -0.148 |
|  |  |  | P | 0.981 | 0.420 | 0.727 | 0.741 |  | 0.521 | 0.035 | 0.001 | 0.120 |
|  |  |  |  |  |  |  |  |  |  |  |  |  |
|  |  | Aβ42 change | r | 0.015 | .207* | 0.037 | .449** |  | -0.043 | -0.097 | -0.055 | -0.092 |
|  |  |  | P | 0.871 | 0.029 | 0.701 | 0.000 |  | 0.651 | 0.310 | 0.563 | 0.332 |
|  |  |  |  |  |  |  |  |  |  |  |  |  |
|  | %Changes | %change in α-synuclein | r | .196* | -0.048 | -0.021 | 0.108 |  | -.255** | 0.025 | -0.040 | 0.100 |
|  |  |  | P | 0.039 | 0.615 | 0.822 | 0.258 |  | 0.007 | 0.793 | 0.678 | 0.296 |
|  |  |  |  |  |  |  |  |  |  |  |  |  |
|  |  | %change in P-tau | r | -0.135 | .545** | -0.048 | 0.162 |  | -0.126 | -.547** | -0.135 | 0.006 |
|  |  |  | P | 0.156 | 0.000 | 0.612 | 0.088 |  | 0.187 | 0.000 | 0.155 | 0.949 |
|  |  |  |  |  |  |  |  |  |  |  |  |  |
|  |  | %change in T-tau | r | 0.128 | -0.079 | 0.153 | -0.002 |  | 0.082 | -0.151 | -.193* | -0.100 |
|  |  |  | P | 0.177 | 0.405 | 0.106 | 0.980 |  | 0.388 | 0.111 | 0.041 | 0.295 |
|  |  |  |  |  |  |  |  |  |  |  |  |  |
|  |  | %change in Aβ42 | r | -0.007 | .224* | 0.018 | .429** |  | -0.071 | -0.100 | -0.070 | -0.103 |
|  |  |  | P | 0.943 | 0.017 | 0.850 | 0.000 |  | 0.455 | 0.292 | 0.460 | 0.279 |
| 2 years | Values | α-synuclein | r |  | 0.055 | .681** | .289** |  | .805** | .349** | .665** | .342** |
|  |  |  | P |  | 0.566 | 0.000 | 0.002 |  | 0.000 | 0.000 | 0.000 | 0.000 |
|  |  |  |  |  |  |  |  |  |  |  |  |  |
|  |  | P-tau | r | 0.055 | 1.000 | .252** | .283** |  | 0.091 | 0.134 | .247** | -0.074 |
|  |  |  | P | 0.566 | 0.000 | 0.007 | 0.003 |  | 0.340 | 0.159 | 0.009 | 0.436 |
|  |  |  |  |  |  |  |  |  |  |  |  |  |
|  |  | T-tau | r | .681** | .252** | 1.000 | .312** |  | .716** | .506** | .923** | .332** |
|  |  |  | P | 0.000 | 0.007 | 0.000 | 0.001 |  | 0.000 | 0.000 | 0.000 | 0.000 |
|  |  |  |  |  |  |  |  |  |  |  |  |  |
|  |  | Aβ42 | r | .289** | .283** | .312** | 1.000 |  | .289** | 0.127 | .290** | .740** |
|  |  |  | P | 0.002 | 0.003 | 0.001 | 0.000 |  | 0.002 | 0.183 | 0.002 | 0.000 |
|  |  |  |  |  |  |  |  |  |  |  |  |  |
|  | Changes | α-synuclein change | r | .354** | 0.028 | 0.076 | 0.063 |  | -0.174 | -0.025 | -0.010 | 0.102 |
|  |  |  | P | 0.000 | 0.771 | 0.428 | 0.511 |  | 0.066 | 0.795 | 0.914 | 0.282 |
|  |  |  |  |  |  |  |  |  |  |  |  |  |
|  |  | P-tau change | r | -0.096 | .763** | -0.056 | .245** |  | -0.098 | -.450** | -0.064 | -0.142 |
|  |  |  | P | 0.314 | 0.000 | 0.555 | 0.009 |  | 0.303 | 0.000 | 0.505 | 0.137 |
|  |  |  |  |  |  |  |  |  |  |  |  |  |
|  |  | T-tau change | r | -0.010 | -0.034 | 0.111 | 0.129 |  | -0.100 | -0.073 | -.236* | 0.063 |
|  |  |  | P | 0.920 | 0.721 | 0.244 | 0.175 |  | 0.294 | 0.446 | 0.012 | 0.511 |
|  |  |  |  |  |  |  |  |  |  |  |  |  |
|  |  | Aβ42 change | r | 0.065 | .517** | 0.089 | .545** |  | 0.049 | -0.134 | 0.023 | -0.069 |
|  |  |  | P | 0.499 | 0.000 | 0.353 | 0.000 |  | 0.611 | 0.160 | 0.808 | 0.470 |
|  |  |  |  |  |  |  |  |  |  |  |  |  |
|  | %Changes | %change in α-synuclein | r | .330** | -0.006 | 0.051 | 0.038 |  | -.205* | -0.039 | -0.039 | 0.090 |
|  |  |  | P | 0.000 | 0.948 | 0.596 | 0.689 |  | 0.030 | 0.687 | 0.682 | 0.346 |
|  |  |  |  |  |  |  |  |  |  |  |  |  |
|  |  | %change in P-tau | r | -0.174 | .733** | -0.115 | .200* |  | -.190* | -.498** | -0.128 | -0.175 |
|  |  |  | P | 0.067 | 0.000 | 0.227 | 0.035 |  | 0.045 | 0.000 | 0.179 | 0.064 |
|  |  |  |  |  |  |  |  |  |  |  |  |  |
|  |  | %change in T-tau | r | 0.031 | -0.030 | 0.137 | 0.130 |  | -0.061 | -0.056 | -.216* | 0.077 |
|  |  |  | P | 0.745 | 0.753 | 0.151 | 0.171 |  | 0.524 | 0.556 | 0.022 | 0.419 |
|  |  |  |  |  |  |  |  |  |  |  |  |  |
|  |  | %change in Aβ42 | r | 0.059 | .494** | 0.082 | .509** |  | 0.035 | -0.154 | 0.016 | -0.098 |
|  |  |  | P | 0.535 | 0.000 | 0.393 | 0.000 |  | 0.711 | 0.105 | 0.870 | 0.302 |

r: Partial correlation coefficient, adjusted for age, sex and disease duration.
